# Two Routes to the Same Equation: Kinetic and Statistical-Thermodynamic Derivations of the Classical Drug-Receptor Occupancy Relationship

**DOI:** 10.7759/cureus.112912

**Published:** 2026-07-18

**Authors:** Robert B Raffa

**Affiliations:** 1 Pharmacology, Temple University, Philadelphia, USA; 2 Pharmacology, University of Arizona, Tucson, USA; 3 Drug Development, Enalare Therapeutics, Mullica Hill, USA

**Keywords:** binding affinity, free energy of binding, grand canonical ensemble, langmuir isotherm, law of mass action, occupancy equation, receptor theory, statistical thermodynamics

## Abstract

The familiar hyperbolic relationship between drug concentration [*D*], drug-receptor dissociation constant (*K*_D_), and fractional receptor occupancy (*p*) expressed as $p = \frac{[D]}{[D] + K_D}$ is the foundation of quantitative pharmacology, tracing to applications of the law of mass action and to adsorption isotherms. Although this kinetic derivation is widely known, it leaves the physical origin of the equilibrium dissociation constant largely unexplained: *K*_D_ is usually represented as an empirical ratio of rate constants rather than a quantity with fundamental thermodynamic meaning. Here we present, in a narrative review conceptual synthesis, a second, independent derivation leading to the same equation, but from equilibrium statistical thermodynamics, treating a receptor as a two-state system exchanging ligand with a bulk drug reservoir within the grand canonical ensemble. This approach reproduces the occupancy equation exactly and reveals *K*_D_ to be a Boltzmann factor, so that ‘affinity’ (1/*K*_D_) is fundamentally a free energy of binding that can be decomposed into enthalpic and entropic contributions. It is shown that the kinetic and thermodynamic approaches are linked by the principle of detailed balance, and that the same partition-function mathematics underlies adsorption isotherms. Beyond its pedagogical value, grounding *K*_D_ in a standard free energy of binding clarifies why affinity differences are not linearly, but exponentially, related to binding energy, and supplies a unifying physical basis for receptor pharmacology, surface adsorption, and ligand-gated molecular switches.

## Introduction and background

Quantitative pharmacology rests on the premise, first articulated by Langley [1] and developed experimentally by Hill [2] and Clark [3], that many drugs must combine with a constituent of a tissue - a “receptor” - in order to produce an effect [4]. Clark's 1926 study of the action of acetylcholine on frog rectus abdominis muscle demonstrated that the relationship between drug concentration and the fraction of receptors occupied follows a simple hyperbola [3], and Clark explicitly recognized that this relationship was mathematically identical to the adsorption isotherm that Langmuir had derived for gas molecules adsorbing onto a fixed number of sites on a solid surface [5] (for convenience, a Glossary of Terms is provided in the Appendices). 

The classical drug-receptor occupancy relationship is:

$p = \frac{[D]}{[D] + K_D}$ (Equation 1).

Here, [*D*] is the drug concentration, *p* denotes the fraction of the total receptor population that is bound by drug at any instant - the fractional receptor occupancy - and *K*_D_ is the equilibrium dissociation constant, in classical theory operationally defined as the drug concentration at the receptor site at which half the receptors are occupied (*p* = 0.5). This equation remains the starting point for analyzing binding assays, estimating *EC*_50_ (the concentration that produces 50% of maximal effect) and *IC*_50_ (the concentration that produces 50% of maximal inhibitory effect) values, and interpreting Schild plots [6], a fuller account of which is given by Colquhoun [7]. The conventional derivation of Equation 1 is purely kinetic: that is, it follows from the law of mass action applied to the reversible reaction between drug and receptor, and the dissociation constant *K*_D_ emerges simply as the ratio of two rate constants. This derivation is mechanistically insightful but conceptually incomplete, since it does not account for why *K*_D_ takes the particular value it does for a given drug-receptor pair, nor does it account for any connection to the thermodynamic enthalpic and entropic quantities involved in binding strength.

The aim here is to show that Equation 1 can be derived through a second, entirely independent route, starting not from reaction kinetics, but from the equilibrium statistical thermodynamics of a two-state receptor exchanging ligand with a bulk reservoir. This route reproduces Equation 1, while also revealing *K*_D_ to be a Boltzmann factor [8] tied directly to a standard free energy of binding [9]. The two derivation routes are united through the principle of detailed balance [10], and the result is therefore part of a broader context including Langmuir adsorption [5] and statistical models of cooperative binding such as Pauling's treatment of hemoglobin [11]. What this thermodynamic view does, and does not, explain about drug action is discussed at the end.

This is a narrative conceptual review of derivations leading to the foundational classical drug-receptor occupancy relationship. Applications to extensions to account for phenomena that strict occupancy theory cannot explain (mentioned in the limits at the end) are beyond the intended scope.

## Review

Kinetic derivation: the law of mass action

Consider a drug D binding reversibly to a receptor R to form a drug-receptor complex DR:

$\mathrm{D} + \mathrm{R} \rightleftharpoons \mathrm{DR}$ (Equation 2),

with forward (association) rate constant *k*_1_ and reverse (dissociation) rate constant *k*_-1_ (or *k*_2_). At equilibrium, the forward and reverse fluxes are equal,

$k_1[D][R] = k_{-1}[DR]$ (Equation 3),

which defines the equilibrium dissociation constant

$K_D = \frac{k_{-1}}{k_1} = \frac{[D][R]}{[DR]}$ (Equation 4).

**Figure 1:**
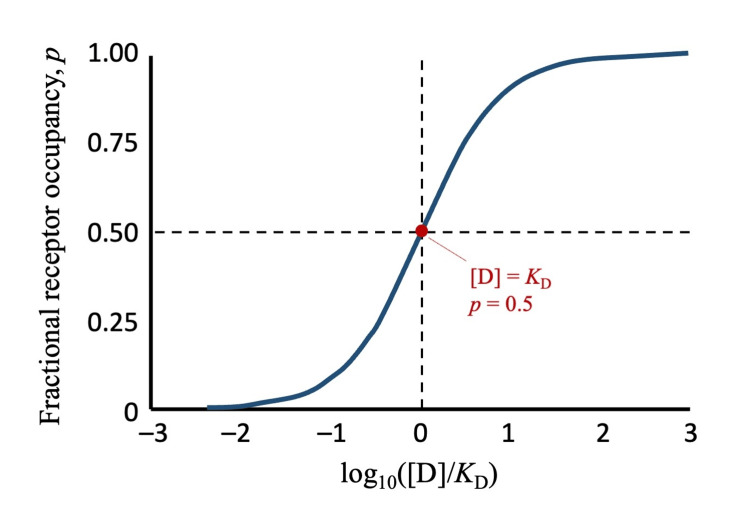
Fractional receptor occupancy as a function of drug concentration. The curve plots Equation 1, $p = \frac{[D]}{[D] + K_D}$, against log_10_([*D*]/*K*_D_). Occupancy is 50% when drug concentration equals *K*_D_, by definition. The curve is sigmoidal when plotted against the logarithm of concentration (as shown), but hyperbolic when plotted against linear concentration. It is identical in form whether derived from the kinetic mass-action argument or the statistical-thermodynamic grand canonical argument.

Writing the total receptor concentration as $[R_t] = [R] + [DR]$ and solving for the fractional occupancy, $p = \frac{[DR]}{[R_t]}$, recovers Equation 1. This is the form Clark used [3], by analogy, from Langmuir's adsorption isotherm [5]; the same hyperbola also describes Michaelis-Menten enzyme kinetics [12], a coincidence of mathematics rather than mechanism. When [*D*] = *K*_D_, *p* = 0.5, which is the operational definition of *K*_D_: the concentration that occupies half the receptor population (Figure 1).

Clark went further and proposed that the magnitude of effect, *E*, is directly proportional to occupancy [13],

$E = E_{\max} \, p = E_{\max} \frac{[D]}{[D] + K_D}$ (Equation 5),

where *E*_max_ is maximal effect. This additional simplification - that binding and effect are the same thing, scaled by a constant - was useful, but shown to be inadequate for many agonists by Ariëns [14] and Stephenson [15], who each introduced a second, independent parameter (called “intrinsic activity” and “efficacy”, respectively) to separate the strength of binding from the magnitude of the response it produces.

What the kinetic derivation leaves unexplained

Equation 4 defines *K*_D_ as a ratio of rate constants, *k*_-1_/*k*_1_. This is mechanistically correct, but it gives no reason why this ratio should take any particular value and no link to the hydrogen bonds, electrostatic contacts, van der Waals packing, or solvent reorganization that medicinal chemists manipulate when they try to improve a compound's ‘affinity’ for a receptor. A first step toward such a link is purely macroscopic. At equilibrium, the chemical potentials μ (the energy stored in chemical bonds that can be released or absorbed when these bonds break or new ones form) of the drug, the receptor, and the drug-receptor complex in Equation 2 must balance

$\mu_D + \mu_R = \mu_{DR}$ (Equation 6),

and, for dilute solutions, $\mu_i = \mu_i^\circ + RT \ln\left(\frac{[i]}{c^\circ}\right)$, where [i] is a chemical species and c° is the standard concentration of i. Substituting into Equation 6 and rearranging gives

$K_D = \exp\left(\frac{\Delta G^\circ}{RT}\right)$ (Equation 7),

where R is the gas constant, T is temperature (in Kelvin), and $\Delta G^\circ = \mu_D^\circ + \mu_R^\circ - \mu_{DR}^\circ$ is the standard free energy of binding. Equation 7 supplies a thermodynamic meaning for *K*_D_ that Equation 4 lacks. But it is still, in an important sense, a black box: it asserts that equilibrium forces an exponential relationship between *K*_D_ and Δ*G*° without explaining the underlying mechanism. For that, statistical mechanics is needed [16].

A statistical-thermodynamic derivation: the receptor as a two-state system

**Figure 2:**
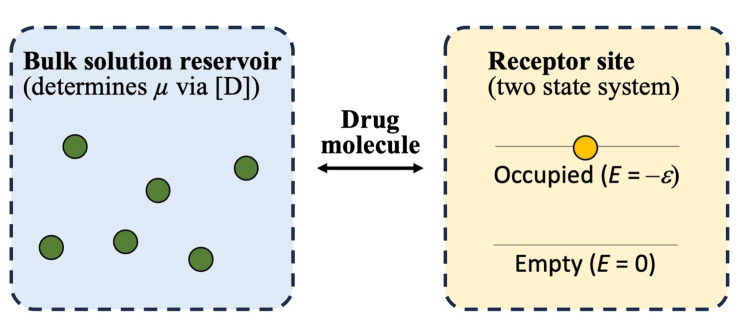
The grand canonical two-state receptor model. A single receptor site (right) toggles between an empty state (energy 0) and an occupied state (energy −*ε*), while exchanging ligand molecules with a bulk-solution reservoir (left) whose chemical potential *μ* is fixed by the drug concentration [*D*]. Weighting each state by the grand canonical Boltzmann factor exp[−β(E − μN)] and substituting $\mu = \mu^\circ + k_B T \ln\left(\frac{[D]}{c^\circ}\right)$ leads to Equation 10. Created by the author using PowerPoint (Microsoft Corporation, Redmond, WA, USA); no artificial intelligence used.

This style of argument is standard in statistical biophysics; see Hill's classic treatment of binding and titration problems [17,18], its extension to cooperative biological systems, and the modern textbook accounts by Wyman & Gill [19] and by Phillips et al. [20]. As a simple case, a single receptor is treated as a system with exactly two accessible states (for an early exposition of application to drug action, see Leff [21]): empty, with zero bound ligands and reference energy *E* = 0; and occupied, with one bound ligand and energy *E* = −*ε*, where *ε* > 0 is the favorable binding energy (Figure 2). But the receptor is not isolated, so it continually exchanges ligand molecules with the surrounding bulk solution, which acts as a reservoir held at a fixed chemical potential set by the bulk drug concentration [*D*].

Because the number of bound ligands fluctuates between 0 and 1, the appropriate description is the grand canonical ensemble (an open system in thermal and chemical equilibrium with a reservoir, allowing the system to exchange both energy and particles), in which each state is weighted by exp[−β(E − μN)], with β = 1/kBT. The grand partition function, *Z*, for the two states is thus

$Z = 1 + e^{\beta(\mu + \varepsilon)}$ (Equation 8).

The probability that the site is occupied is the Boltzmann weight (the relative probability of a system occupying a state) of the occupied state divided by *Z*:

$p = \frac{e^{\beta(\mu + \varepsilon)}}{1 + e^{\beta(\mu + \varepsilon)}}$ (Equation 9).

Equation 9 already has the form of a Fermi-Dirac-type occupation probability (that the transition rate between states of limited occupancy is proportional not only to the occupancy of the initial state, but also to the “emptiness” or availability of the final state); the remaining step is to translate the chemical potential, *μ*, into the quantity measured, namely the bulk concentration [*D*]. For an ideal dilute solution (where the drug concentration is sufficiently low that drug molecule interactions are negligible, while the solvent molecules remain surrounded primarily by other solvent molecules, mimicking the pure liquid state),

$\mu = \mu^\circ + k_B T \ln\left(\frac{[D]}{c^\circ}\right)$ (Equation 10).

Substituting Equation 10 into Equation 9 and defining $K_D \equiv c^\circ \exp\!\left[-\beta\left(\mu^\circ + \varepsilon\right)\right]$ - a constant that absorbs every molecule-specific energetic term - gives

$e^{\beta(\mu + \varepsilon)} = \frac{[D]}{K_D}$ (Equation 11).

Therefore, $p = \frac{[D]/K_D}{1 + [D]/K_D} = \frac{[D]}{[D] + K_D}$, which is Equation 1, but now derived without any reference to rate constants. The same grand canonical reasoning, applied to a single quantum state that can hold at most one electron rather than one ligand, yields the Fermi-Dirac distribution; applied to gas molecules and a fixed lattice of surface sites, it yields the Langmuir isotherm [22]. A wide-ranging discussion of this style of reasoning across molecular and cell biology is given by Phillips [23].

Affinity as a free energy

Equation 7 can be rearranged to give $\Delta G^\circ = RT \ln K_D$, so

$\mathrm{affinity} = \frac{1}{K_D} = \exp\left(-\frac{\Delta G^\circ}{RT}\right)$ (Equation 12).

Therefore, affinity is, fundamentally, an exponential function of a standard free energy of binding [24]. At 37 °C, *RT* ln 10 ≈ 1.4 kcal mol^-1^; each additional 1.4 kcal mol^-1^ of binding free energy therefore corresponds to a roughly ten-fold gain in affinity, something that medicinal chemists track while trying to optimize a lead compound. The free energy itself separates into enthalpic and entropic contributions,

$\Delta G^\circ = \Delta H^\circ - T\Delta S^\circ$ (Equation 13).

Enthalpic terms arise from specific molecular contacts (e.g., hydrogen bonds, electrostatic and van der Waals interactions, and the cost of desolvating polar groups), whereas the entropic term reflects a balance between the conformational and translational freedom that the drug and receptor lose on binding (typically unfavorable) and the favorable hydrophobic effect, in which burial of nonpolar surface area releases ordered water into bulk solvent. This explains, for instance, why rigidifying a flexible ligand for the purpose of losing less conformational entropy on binding is a recognized strategy that has been tried for improving affinity even when no new enthalpic contact is added [25].

Reconciling the two derivations: detailed balance

The kinetic derivation defines *K*_D_ as *k*_-1_/*k*_1_; the thermodynamic derivation defines the same symbol as exp(Δ*G*°/R*T*). These are not two competing facts, but two faces of one constraint: the principle of detailed balance requires that, for a reaction at thermodynamic equilibrium, the ratio of forward and reverse rate constants equals the Boltzmann factor of the free-energy difference between products and reactants,

$\frac{k_1}{k_{-1}} = e^{-\Delta G^\circ/RT}$ (Equation 14).

Kinetics and thermodynamics are therefore not independent: kinetics describes how quickly a drug-receptor system reaches equilibrium (a question of diffusion, conformational search, transition-state barriers, etc.), whereas thermodynamics describes where that equilibrium lies. Equation 14 shows that the two routes converge on the same occupancy equation.

A universal equation and its limits

The grand canonical argument is indifferent to the physical identity of the particle being exchanged. Applied to gas molecules adsorbing onto a fixed population of surface sites, it is the Langmuir isotherm [5]; applied to a single-particle energy level that can hold at most one electron, it is the Fermi-Dirac distribution; and in one of the most influential applications of statistical mechanics to biology, Pauling used essentially this reasoning, generalized to four interacting sites, to model the cooperative binding of oxygen to hemoglobin [26]. The recurrence of similar mathematics across adsorption physics, electronic structure, and ligand pharmacology is not a coincidence, but a reflection of a shared underlying mechanistic model - a small number of discrete sites in diffusive contact with a particle reservoir - a point developed in the context of cellular and molecular biology generally.

Some limits of the present analysis deserve emphasis. First, both derivations concern only the binding equilibrium captured by Equation 1; neither says anything about Equation 5, Clark's separate assumption that effect is proportional to occupancy, an assumption now known to fail for partial agonists and for systems exhibiting a receptor reserve, where near-maximal response can be produced at well below saturating occupancy. Ariëns [14] and Stephenson [15] each addressed this by introducing efficacy as a parameter independent of affinity, and Black & Leff [27] later formalized the distinction in their operational model, which separates a “cognitive” binding step from a “transducer” function relating occupancy to response. Extending the grand canonical treatment to efficacy would require a model with more than two states - for example, distinguishing active and inactive conformations of the occupied receptor - of the kind used in allosteric (Monod-Wyman-Changeux-type) [9] descriptions of cooperative receptors; this is a natural direction for the present framework, but lies outside the present scope. Beyond efficacy, drug-receptor concepts have been productively extended to account for phenomena that cannot adequately be explained by strict occupancy theory. For example, biased agonism - the ability of different ligands at the same receptor to preferentially stabilize receptor conformations that couple to distinct downstream effectors - has been treated formally within multi-state partition-function models, where each active conformation carries its own Boltzmann weight [28,29]. And the identification of Δ*G*° as the operative binding parameter has spurred the development of computational methods, including alchemical free-energy perturbation and end-state (MM-PBSA/GBSA) approaches, for predicting *K*_D_ values from structural data, a direction that has become central to structure-based drug design [30,31].

Second, the thermodynamic derivation explains why *K*_D_ must take the form it does, but it does not, by itself, predict the numerical value of *ε* (or Δ*G*°) for any particular drug-receptor pair; that remains an empirical and, increasingly, a computational question for structural and physical chemistry. What the statistical-mechanical route contributes is a principled answer to the question that the kinetic derivation leaves open: namely, *K*_D_ is not an arbitrary empirical constant, but rather is a Boltzmann factor, and affinity is, irreducibly, a free energy.

## Conclusions

The classical receptor occupancy equation can be reached by two independent routes: a kinetic argument from the law of mass action, in which *K*_D_ is a ratio of rate constants, and a statistical-thermodynamic argument from the grand canonical ensemble, in which the same symbol is a Boltzmann factor of a standard free energy of binding. The principle of detailed balance guarantees that the two must agree. Presenting both derivations side by side has pedagogical value - it shows that a relationship often introduced as an empirical curve-fitting method is in fact required by basic principles of chemical equilibrium - and conceptual value, since it grounds drug-receptor affinity in the same free-energy language used throughout physical chemistry and biophysics, from gas adsorption to hemoglobin to the broader statistical mechanics of the cell.

## Appendices

**Table 1:** Glossary of terms Guides to key terms and concepts represented in the equations and used in the derivations. The symbols follow standard conventions in pharmacology and statistical mechanics.

Term	Definition
Adsorption isotherm	The relationship between the equilibrium amount of substance adsorbed onto a solid surface and its concentration in liquid solutions
Affinity (1/K_D_)	The reciprocal of K_D_; it quantifies the attractive forces between a drug and a receptor.
Boltzmann factor	An exponential term that quantifies the relative likelihood of a system being in a specific energy state at thermal equilibrium at a given temperature.
Canonical ensemble	A statistical-mechanical framework for a system that can exchange both energy and particles with a reservoir held at fixed chemical potential μ and temperature T; the natural description for a receptor site that fluctuates between empty and occupied states.
Chemical potential (μ)	The free energy per molecule of a chemical species in solution; it determines the thermodynamic “pressure” driving ligand association with a receptor.
Detailed balance	The principle that at thermodynamic equilibrium every elementary process and its reverse occur at equal rates; guarantees that the ratio of forward and reverse rate constants equals the Boltzmann factor of the free-energy difference.
EC_50_	The drug concentration that produces 50% of the maximum observed pharmacological effect.
Efficacy (ε)	A parameter that represents how effectively a bound drug activates a receptor, independent of affinity; explains why two drugs with identical K_D_ can produce different maximal responses.
Enthalpy (ΔH°)	The heat contribution to binding, arising from molecular contacts (e.g., hydrogen bonds, electrostatic interactions, van der Waals forces) and desolvation.
Entropy (ΔS°)	The “degree of disorder” contribution to drug-receptor binding; it is typically unfavorable due to loss of conformational and translational freedom on binding; partly offset by the hydrophobic effect.
Equilibrium dissociation constant (K_D_)	In classical drug-receptor theory, the drug concentration at which p = 0.5; a concentration-based inverse measure of binding affinity (i.e., a low K_D_ = high affinity).
Fractional receptor occupancy (p)	The proportion of the total receptor population that is occupied by a drug molecule at equilibrium; it ranges from 0 (unoccupied) to 1 (fully occupied).
Grand partition function (X)	The sum of Boltzmann weights, exp[−β(E − μN)], over all accessible states; the generating function from which thermodynamic quantities are derived.
IC_50_	The concentration of drug that results in a 50% inhibition of a defined reference response.
Law of mass action	The observation, or assumption, that the rate of a chemical process is (directly) proportional to the product of the amounts or concentrations of the interacting substances.
Ligand	A term that refers to a generalized molecule that binds to a receptor, independent of the nature of the molecule’s (lack of) action.
Receptor	In pharmacology, a macromolecular target (typically a protein) with which a drug combines to enhance or diminish a biological effect.
Schild plot	A graphical method for quantifying competitive antagonism; the x-axis intercept yields the antagonist equilibrium dissociation constant.
Standard free energy of binding (ΔG°)	The thermodynamic “driving force” for drug–receptor association; it is related to affinity (K_D_) by ΔG° = RT lnK_D_.
